# Tailored Bioactive Compost from Agri-Waste Improves the Growth and Yield of Chili Pepper and Tomato

**DOI:** 10.3389/fbioe.2021.787764

**Published:** 2022-01-24

**Authors:** Asma Imran, Fozia Sardar, Zabish Khaliq, Muhammad Shoib Nawaz, Atif Shehzad, Muhammad Ahmad, Sumera Yasmin, Sughra Hakim, Babur S. Mirza, Fathia Mubeen, Muhammad Sajjad Mirza

**Affiliations:** ^1^ Soil and Environmental Biotechnology Department, National Institute for Biotechnology and Genetic Engineering (NIBGE), Faisalabad, Pakistan; ^2^ Department of Biology, Missouri State University, Springfield, MO, United States

**Keywords:** multi-plant waste compost, bioactive compost, FESEM, fish, MPN-PCR, chili, tomato

## Abstract

An extensive use of chemical fertilizers has posed a serious impact on food and environmental quality and sustainability. As the organic and biofertilizers can satisfactorily fulfill the crop’s nutritional requirement, the plants require less chemical fertilizer application; hence, the food is low in chemical residues and environment is less polluted. The agriculture crop residues, being a rich source of nutrients, can be used to feed the soil and crops after composting and is a practicable approach to sustainable waste management and organic agriculture instead of open-field burning of crop residues. This study demonstrates a feasible strategy to convert the wheat and rice plant residues into composted organic fertilizer and subsequent enrichment with plant-beneficial bacteria. The bioactive compost was then tested in a series of *in vitro* and *in vivo* experiments for validating its role in growing organic vegetables. The compost was enriched with a blend of micronutrients, such as zinc, magnesium, and iron, and a multi-trait bacterial consortium AAP (*Azospirillum*, *Arthrobacter*, and *Pseudomonas* spp.). The bacterial consortium AAP showed survival up to 180 days post-inoculation while maintaining their PGP traits. Field emission scanning electron microscopic analysis and fluorescence *in situ* hybridization (FISH) of bioactive compost further elaborated the morphology and confirmed the PGPR survival and distribution. Plant inoculation of this bioactive compost showed significant improvement in the growth and yield of chilies and tomato without any additional chemical fertilizer yielding a high value to cost ratio. An increase of ≈35% in chlorophyll contents, ≈25% in biomass, and ≈75% in yield was observed in chilies and tomatoes. The increase in N was 18.7 and 25%, while in P contents were 18.5 and 19% in chilies and tomatoes, respectively. The application of bioactive compost significantly stimulated the bacterial population as well as the phosphatase and dehydrogenase activities of soil. These results suggest that bioactive compost can serve as a source of bioorganic fertilizer to get maximum benefits regarding vegetable yield, soil quality, and fertilizer saving with the anticipated application for other food crops. It is a possible win-win situation for environmental sustainability and food security.

## 1 Introduction

An exponential increase in the global population demands sustainability, safety, and security of food with minimum burden on the economy, Earth, and the environment. An input-intensive conventional farming, however, ensures food safety but leaves a long-term harmful impact on the production system, food quality, environmental sustainability, biodiversity, greenhouse gas emission, and human health. In the last 2 decades, concerted efforts have been made for exploring ways for the sustainable future food security with a lesser reliance on chemicals and to achieve the United Nations Sustainable Development Goals (SDGs) regarding sustainable life and environment (UNDP, 2021). Organic farming is a natural way of crop production that involves the use of ecologically safe crop-fertilization and pest-management strategies such as compost, green manure, biological fertilizers, or biopesticides. The incorporation of organic fertilizer, for example, compost developed from farm waste (crop residues, animal waste, *etc*.), into the soil replenishes the soil with plant nutrients that promise higher yields of subsequent crops (Gupta et al., 2007).

A huge amount of crop residues are wasted annually either by burning in the field (after harvest) or in the industry during the refining process (husk and bran) (Abbas et al., 2012). Around 40% of N, 35% P, 85% K, and 45% S taken up by the rice plants remain in vegetative parts (Dobermann and Fairhurst, 2002), which can be reused to nourish soil and plants (Tahir et al., 2006) or reutilized in the industry (Calabi–Floody et al., 2018). The burning causes a complete loss of N, 25% of P, 20% of K, and up to 60% of S (Dobermann and Fairhurst, 2002), and is a major contributor of air pollution and greenhouse gases (Udeigwe et al., 2015; Romasanta et al., 2017; Andini et al., 2018). Under the field condition, the degradation of straw is very slow, and crop impact is less positive because it is chemically stable yielding a high value to cost ratio and contains high lignocellulosic material with a high C: N ratio (Dobermann and Fairhurst, 2002; Chen, 2014). Composting is a microbial-driven process that accelerates the waste degradation and conversion of complex materials into usable, simpler organic and inorganic forms (Zhang et al., 2016; Bhattacharjya et al., 2021).

Composted organic fertilizers developed from farm waste significantly improve soil carbon status, nutrient balance, and overall growth and yield of plants (Das et al., 2003; Whitbread et al., 2003; Ye et al., 2020; Sharma et al., 2021). Having relatively lower ratios of nutrients than chemical fertilizers, the field application rate of compost is very high (t ha^−1^) that makes it not only impracticable but also economically expensive to apply in large agricultural fields or organic agriculture. The problem can be solved by improving the quality and effectiveness of the compost either by enrichment with essential nutrients (bioactive compounds) or indirectly (microbes). Bioorganic fertilizer (BOF) combines the benefits of bacteria and the organic matter, and is more effective than the microorganisms alone or the organic matter (Li et al., 2021). Bioactive compounds or microbes stimulate various biological processes and exert direct positive impact on the plant. The addition of plant growth-promoting bacteria (PGPB) to the compost makes it biologically active and effective for seed germination and plant growth, soil rehabilitation, and disease suppression (Tahir et al., 2006; El-Akshar et al., 2016; Kaur et al., 2019). The phytohormone-producing PGPB mediate water and nutrient uptake due to increased root proliferation that ultimately improve plant growth and yield (Imran et al., 2021). The effectiveness of bioactive compost, however, depends upon the survival and physiological efficiency of microbes (Altaf et al., 2014) along with the organic contents and moisture-holding capacity of the compost (Mahdi et al., 2010; Calabi–Floody et al., 2019). However, the individual impacts of compost or PGPR on plant growth are well-documented, but the synergistic potential of BOF has only been reported in a few plants such as wheat (Akhtar et al., 2009; Kanwal et al., 2017), cotton (Zewail and Ahmed, 2015), sunflower (Arif et al., 2017), cucumber (Nadeem et al., 2017), and potato (Li et al., 2021) on a pot scale level.

Recent estimates show that global staple cereal production will increase 50% by 2050 (FAO and UNICEF, 2017), which will constantly require a huge amount of chemical fertilizer inputs on the one hand, and generate massive crops residues (e.g., straw, bran, and husk) on the other hand. Composting these crop residues and subsequent soil application will generate a sustainable organic agriculture system which will have minimum reliance on chemical inputs and crop waste burning (Liang et al., 2012). In this study, the focus was on the chilies and tomatoes as these are among the main vegetables grown around the globe and were not tested for enriched BOF earlier. Tomatoes are on the top with an estimated production of 180.77 million metric tons per year, and chili production is also on the boom, with an average of 38.03 million metric tons per year (Shahbandeh, 2021). It was hypothesized that enriched BOF will support the plant growth as well as improve soil health. The present study demonstrates the beneficial impact of biologically active compost for growing chilies and tomatoes without additional mineral fertilizer for sustainable management of crop waste, increased agriculture productivity, and safer environment.

## 2 Materials and Methods

### 2.1 Development of Compost

Compost was developed in cemented plots (12 × 5 × 1.2 m) by using wheat and rice crop residues (<2 cm) from experimental fields. Wheat and rice straw were collected, completely dried, and co-composted with nitrogen-rich green plants *Sesbania bispinosa* and *Trifolium alexandrinum* (>2 cm), respectively, in the ratio of 2:1, and *Azolla pinnata* in the ratio of 1:4 for nitrogen enrichment/nitrogen urea (1.0 kg/40 kg compost) during composting to decrease the C: N ratio and speedup composting of raw materials (Rovshandeh et al., 2007). The aerobic composting technique was used to decompose heap (Liu et al., 2011), maintaining proper aeration and moisture level 50–55% by turning/mixing the raw material and the addition of required water after an interval of 15 days. Heap was covered with a plastic sheet to prevent loss of moisture and heat produced. The temperature of the composting heap was taken using a thermometer at a regular interval of 15 days. The composted material was left for stabilization for 1 week, and then finally grounded (2.0 mm) and sieved to ensure homogeneity.

### 2.2 Analysis of Compost Metagenome

DNA was extracted from three replicates of both wheat and rice samples using a DNA Isolation Kit (MP Biomedicals, United States) according to the manufacturer’s instructions. The eluted DNA was further processed for amplicon library construction for Illumina sequencing using a two PCR steps’ approach with two different primer pairs for the V3–V4 region of the 16S rRNA gene as previously described (Hakim et al., 2020). Paired-end Illumina reads were assembled using the Mothur software (Schloss et al., 2009). The sequences where the forward and reverse sequence did not match were filtered out to minimize the effect of random sequencing error. Furthermore, the primer sequences were trimmed, and those of low-quality with read lengths <370 bp, homopolymers > 8 bases, and the sequences with >3 continuous ambiguous bases (i.e., N) were filtered out using Mothur. The high-quality sequences were screened for chimeras and clustered into operational taxonomic units (OTUs) at 97% of sequence similarity using the Ribosomal Database Project (RDP) platform. The sequences have been deposited in GenBank Sequence Read Archive under the Bio project.

### 2.3 Analysis of Compost Quality

The compost was characterized for quality parameters including pH, electrical conductivity, nitrogen and phosphorus content, and organic matter. Compost samples were mixed in water (1:5 ratio w/v) and placed at a shaker for 2 h. The pH and the electrical conductivity (EC) were taken using a pH meter (pH/ion analyzer 350, CORNING) and an EC meter (Multi-range Conductivity Meter Clarkson HI23151, Hanna Instruments, Italy), respectively (Roca–Pérez et al., 2009). The nitrogen content of the compost was determined by following the Kjeldahl method (Sparks et al., 2020), while phosphorus was determined by using the vanadium phosphomolybdate method (Yoshida et al., 1976), followed by taking absorbance through a spectrophotometer (double-beam UV-Vis, Camspec-M350, United Kingdom). Organic matter was quantified as described (Schollenberger, 1945).

### 2.4 Development of Bioactive Compost

#### 2.4.1 Bacterial Consortium

The compost was enriched with a consortium of three PGPR strains, *Azospirillum brasilense* strain ER-20, *Arthrobacter oxydans* WP-2, and *Pseudomonas stutzeri* strain K1 abbreviated as AAP. The strains are well characterized and compatible with each other (Ref). Antibiotic-resistant derivatives of AAP strains were developed for successful and selective recovery from the compost by following the method of Hanif et al. (Hanif et al., 2015). The antibiotic-resistant derivatives of ER-20 and K-1 were developed against streptomycin 400 μg/ml, while WP-2 antibiotic-resistant derivatives were developed against streptomycin 200 μg/ml + rifampicin 20 μg/ml. The mean generation time, effectiveness, and efficiency of mutants were estimated before inoculating them to the compost.

Wild and antibiotic-resistant derivative strains were grown in the LB broth separately; after 24 h, a culture of each strain was centrifuged (4,000 rpm, 4°C for 15 min), and the cells were resuspended in saline to get an OD of 0.45 λ 600 nm.

#### 2.4.2 Nutrients

The micronutrients were added to get a final concentration of Zn: 5 mg Kg^−1^, Mg: 50 mg Kg^−1^, and Fe: 50 mg Kg^−1^. The nutrients were mixed thoroughly in the compost.

For the development of bioactive compost, fully grown bacterial strains (*Azospirillum brasilense* strain ER-20, *Arthrobacter oxydans* WP-2, and *Pseudomonas stutzeri* strain K1) were mixed in the ratio of 1:1:1 to get a consortium AAP. The cell pellet was obtained by centrifugation, resuspended in 500 ml normal saline, and mixed with the compost (100 ml 10^7^ CFU per Kg compost). The nutrients were added during the mixing process. The compost was air-dried and then packed in the bags. The compost inoculated with saline was kept as a control.

### 2.5 Analysis of PGPR Efficacy in Bioactive Compost

#### 2.5.1 Surface Morphology and Bacterial Distribution Using FESEM

Compost surface morphology, distribution, and population of inoculated bacteria were analyzed by FESEM at 30, 60, 90, 120, 150, and 180 dpi. For FESM analysis, 50–100 mg of samples were taken aseptically and dried at room temperature to avoid surface tension artifacts; then the samples were carefully mounted on an aluminum stub using a double stick carbon tape. The stub was washed with acetone and air-dried before sample mounting (Qu et al., 2017). The specimen was focused using coarse, and fine focus was used at 5,000X at 10kv to get the fine image of the sample.

#### 2.5.2 Bacterial Detection Using Fluorescence *In Situ* Hybridization

The inoculated PGPR were detected using FISH at 90 dpi using fixation and hybridization protocol for soil samples (Amann et al., 1990; Stein et al., 2005; Bertaux et al., 2007) with slight modification. One gram of the bioactive compost sample was taken, diluted in 9.0 ml of extraction buffer (0.8 mM MgSO_4_·7H_2_O, 1 mM CaCl_2_·2H_2_O, 1.7 mM NaCl, and 5% Tween-20), and homogenized using a vortex mixer for 5–10 min. After sedimentation for 15 min, 3.0 ml from the supernatant was mixed with three volumes of 4% PFA (paraformaldehyde) and incubated overnight at 4°C. The fixed samples were centrifuged for 5 minutes at 5,000 rpm; the pellet was washed thrice with 1xPBS and resuspended in 1 ml 0.01% toluidine blue for 1 hour. Centrifugation and washing were repeated; the pellet was resuspended in 1:1 PBS ethanol and finally stored at −20°C. Fluorescently labeled oligonucleotide probes used in the FISH analysis are mentioned in Supplementary Table S1. These oligonucleotide probes were synthesized with indocarbocyanine (cy3) and fluorescein-5-isothiocyanate (FITC) at 5′ end (Interactiva Biotechnologie GmbH, Ulm, Germany) (Majeed et al., 2018). Fixed samples were placed on slide wells, air-dried, and dehydrated by washings with 50, 80, and 100% ethanol, respectively (3 min each). A 2-μl oligonucleotide probe and 18 μl hybridization buffer were added to each well; compost samples were treated with hybridization buffer and 15 pmol of each FLUOS-labeled EUB 338 specific for bacteria and Cy3-labeled probe GAM42a specific for gamma Proteobacteria. After 3 h of hybridization at 46°C, samples were treated with a washing buffer for 20 min, and then washed with sterilized water, air-dried, shifted on a microscopic slide in Citifluor (mounting buffer), and observed on CLSM (Olympus FV 1000, Japan). Pure bacterial strains were also grown, and their cells were fixed, hybridized, washed, air-dried, and observed as described above (Amann et al., 1995).

#### 2.5.3 Viability and Efficiency of PGPR

Survival of AAP consortium inoculated to the compost was analyzed at 7, 15, 30, 60, and 90 dpi using a standard serial dilution plating technique (Mislivec and Bruce, 1977; Somasegaran and Hoben, 1994). Wild strains were recovered on simple LB-agar plates, while antibiotic-resistant derivatives were recovered on LB-antibiotic selection media. The bacterial population from control (mock) bags was recovered on LB agar plates. Plates were incubated at 28°C for 24–48h until the appearance of colonies. The viable cells were calculated by counting CFU ml^−1^ at each interval on a colony counter and converted to log values.

Recovered colonies at each time were tested for their effectiveness as PGPR. P-solubilization was checked by spot inoculation on Pikovskaya’s plate. The formation of the halo zone was confirmed each time and compared to pure culture (Pikovskaya, 1948). Similarly, IAA production was tested using the colorimetric method and compared to pure culture (Gordon and Weber, 1951). Nitrogen fixation was also detected by inoculation into the NFM (nitrogen-free malate) semisolid medium *via* the acetylene reduction assay (ARA) as described earlier (Mirza et al., 2001). Furthermore, 100 µL of the sample was taken from dilutions 1, 2, and 3 and added to the NFM semisolid medium at every time interval to calculate the most probable number (MPN) (Alexander, 1965).

#### 2.5.4 PCR-Based Detection of Bacteria

Samples were collected in three replicates from both control (non-inoculated) as well as bioactive (inoculated) compost after three and 6 months. The DNA was extracted from 0.5 g compost samples using the Fast Prep soil DNA Spin Kit, and 10-fold serial dilutions of DNA were prepared for MPN–PCR. These dilutions along with original DNA from the pure culture of K-1 were used in MPN–PCR. Strain-specific primers (Mirza et al., 2006) were used for MPN–PCR-based detection of inoculated strain K-1 in the bioactive compost. PCR reaction and amplification conditions were the same as previously described (Mirza et al., 2006).

### 2.6 Plant Testing of Compost and Bioactive Compost

#### 2.6.1 Experiment 1: Initial Testing of Wheat and Rice Compost and PGPR on Chili in Pots

This experiment was set up in a completely randomized design (CRD) with six treatments and three replicates each using rice-straw compost (RSC) and wheat-straw compost (WSC) separately. The bacterial inoculum used was AAP consortium. The treatments’ details are as follows: T1 = Control soil + chemical fertilizer (CF), T2 = AAP-inoculated soil (B), T3 = RSC (CR), T4 = AAP-inoculated RSC (CRB), T5 = WSC (CW), and T6 = AAP-inoculated WSC (CWB). The soil and compost were thoroughly mixed by sieving three times in a ratio of 1:3 (v/v) and filled into the pots (8 inches dia and 9 inches depth). Three chilies’ seedlings (Hybrid; Golden Hot) were transplanted in each pot. Pots were irrigated with tap water whenever required. Harvesting was done after maturity and plant growth parameters, that is, root length, shoot length, fresh weight, dry weight, number of chilies, fresh weight, and dry weight of chilies, were recorded at the time of harvesting.

#### 2.6.2 Experiment 2: Testing of Wheat: Rice Compost Combination With PGPR for Chilies’ Growth in Pots

After validation of the beneficial impact of wheat and rice straw composts and PGPR efficacy, a 1:1 combination rice-straw compost (RSC) and wheat-straw compost (WSC) was developed for enrichment with PGPR for further experiments. Bioactive compost and soil were thoroughly mixed in a 1:3 ratio (v/w) and passed through a 2-mm sieve, and then earthen pots (8-inch diameter and 9-inch depth) were filled with this mixture. Experimental set up includes three treatments: T1 = Control soil + chemical fertilizer (CF), T2 = Compost (C; RSC: WSC 1:1 v/v), and T3 = PGPR inoculated compost (CB; RSC: WSC 1:1 v/v + AAP). The experiment was laid out in the CRD using conditions similar to those of the abovementioned experiment.

#### 2.6.3 Experiment 3: Testing of Bioactive Wheat: Rice Compost Combination for Chilies’ Growth in Microplots

After validation of the results of pot experiment 2, the same treatments were validated in the microplots. The experimental set up includes same three treatments: T1 = Control soil + chemical fertilizer (CF), T2 = Compost (C; RSC: WSC 1:1 v/v), and T3 = PGPR inoculated compost (CB; RSC: WSC 1:1 v/v + AAP). The experiment was laid out in the RCBD using conditions similar to those the above-mentioned experiment in the chili growing season.

### 2.7 Experiment 4: Effect of Bioactive Compost on Chilies and Tomato plants in the Mini Tunnel

Plant experiments were conducted for 2 years to evaluate the effect of bioactive compost on chilies and tomatoes during the year 2018 and 2019. There treatments were classified as T1 = Control soil + chemical fertilizer (CF), T2 = simple compost (CS), T3 = nutrient-enriched compost (CN; RSC: WSC 1:1 v/v + FMZ), and T4 = Bioactive compost (BAC; RSC: WSC 1:1 v/v-AAP inoculated + FMZ). The experiment was carried out in the RCBD. Sowing of hybrid chili variety (Royal Hot) nursery was done on 15th October, while transplanting was done on 15th December on plot size = 3 m × 1 m (mini tunnel) with five replicates for each treatment. Tomato (variety Nadir) nursery sowing was done on 30th October, while transplantation was done on 20th December on a plot size of plot size: 3 × 1 m (mini tunnel) with five replicates for each treatment. The plant-to-plant distance was maintained at 25 cm. The compost was thoroughly mixed (1:3 v/v) in the 10–20 cm topsoil of the plot. Irrigation was done with tap water whenever required. Harvesting was done at 60, 90, 120, and 130 days post-transplantation (DPT). Data were recorded for morphological, physiological, and yield-related plant parameters. For morphological parameters, three plants from each replicate were uprooted, and mean shoot/root length and fresh/dry weights were recorded. For yield parameters, data were recorded from five plants from each replicate, and the mean was calculated.

#### 2.7.1 Survival of Inoculated Bacteria in the Rhizosphere

For the detection and survival of bacteria inoculated to bioactive compost, PCR and FISH analyses were performed. The roots samples were carefully taken from different treatments, the total DNA was extracted from the rhizosphere 0.5 g soil using the Fast Prep soil DNA Spin Kit, and 10-fold serial dilutions of DNA were prepared for PCR. These dilutions along with original DNA from the pure culture of K-1 were used in PCR as described (Mirza et al., 2006). Similarly, the root samples were fixed and processed for FISH analysis as described (Majeed et al., 2018).

#### 2.7.2 Analysis of Photosynthetic Efficiency

Different leaf photosynthetic parameters, that is, transpiration rate, stomatal diffusive resistance, leaf temperature, quantum, and relative humidity, were measured by using a leaf porometer (L1-1600 L1-COR USA). The porometer was attached to one side of broad leaves exposing the other surface to the ambient air to allow the energy emission by the leaf through radiation. Different parameters were calculated as the standard protocol. Chlorophyll a and b were determined using 500 mg fresh leaf extracted overnight with 80% acetone and centrifuged at 10,000 ×g for 5 min. The absorbance of the supernatant was estimated using a spectrophotometer at 480-, 645-, and 663-nm wavelengths against the solvent, and chlorophyll contents were calculated (Armon, 1949).

#### 2.7.3 Analysis of Fruit Nutrient Contents

The data regarding total N contents in tomato and chili fruits were estimated using the Kjeldahl method (Keeney and Nelson, 1982), and total P contents were estimated using the vanadium phosphomolybdate method (Twine and Williams, 1971).

#### 2.7.4 Soil Enzyme Activities

After plant harvesting, the rhizosphere soil was analyzed for alkaline phosphatase and dehydrogenase enzyme activity in response to different compost treatments. Alkaline phosphatase activity was analyzed as described by Kramer and Yerdei (1959). 1.0 g soil sample was mixed into 5.0 ml of 0.5% disodium phenyl phosphate followed by the addition of 0.2 ml toluene and incubated on a shaker at 37°C for 2 h. The soil suspension was filtered, 1 ml of filtrate in 4 ml water was mixed with 4 ml of borate buffer (0.05 ml, pH = 10 + 0.5 ml 2% 4-Amino antipyrine +0.5 ml 8% potassium ferrocyanide). Then incubation was performed at room temperature for 1 h, and the change from yellowish to red was observed. The optical density of the supernatant was measured at 510 nm on a spectrophotometer (UV-1201, Shimadzu Crop, Japan). The blank solution consists of water and reagent in a 1:1 ratio. 1,000 ppm standard stock of phenol was prepared by dissolving 1 g of phenol in up to 1,000 ml water. From the stock solution, various standards of 0.3, 0.5, 0.8, 1, 1.5, and 1.8 were prepared and a standard curve was plotted, and the equation was generated to calculate phosphatase activity of soil samples.

Soil dehydrogenase activity was measured using the method developed by Klein et al. (1971). 2.0 g soil was incubated with 2 ml of a 1% triphenyl tetrazolium chloride (TTC) solution in 0.1 M Tris HCl buffer (pH = 7.4) at 37°C for 24 h. Then 10 ml methanol was added and kept for 30 min at 37°C for the development of reddish-orange color (conversion of triphenyl tetrazolium chloride (TTC) to triphenyl formazan (TF)) followed by the measurement of optical density at 485 nm using a spectrophotometer (UV-1201, Shimadzu Crop, Japan). A blank solution was prepared using 2 ml of 1% TTC solution in 10 ml methanol as described above, while triphenyl formazan (TF) standards of 2, 10, 20, 30, 50, and 100 ppm were used to draw a standard curve for the quantification of soil dehydrogenase activity.

#### 2.7.5 Total Soil Bacterial Count

The number of bacteria present in the rhizosphere of all the treatments was counted using standard colony-forming units (CFUs) by a serial dilution plating technique using the LB medium as described (Somasegaran and Hoben, 1994).

### 2.8 Statistical Analysis

The data were subjected to analysis of variance (ANOVA) using M state software, and significance was measured at LSD 0.05. Graphs were constructed using Microsoft Excel (2019) or Slide Write (7.0), and assembled using CorelDraw (R 12).

## 3 Results

### 3.1 Analysis of Compost Metagenome

**FIGURE 1:**
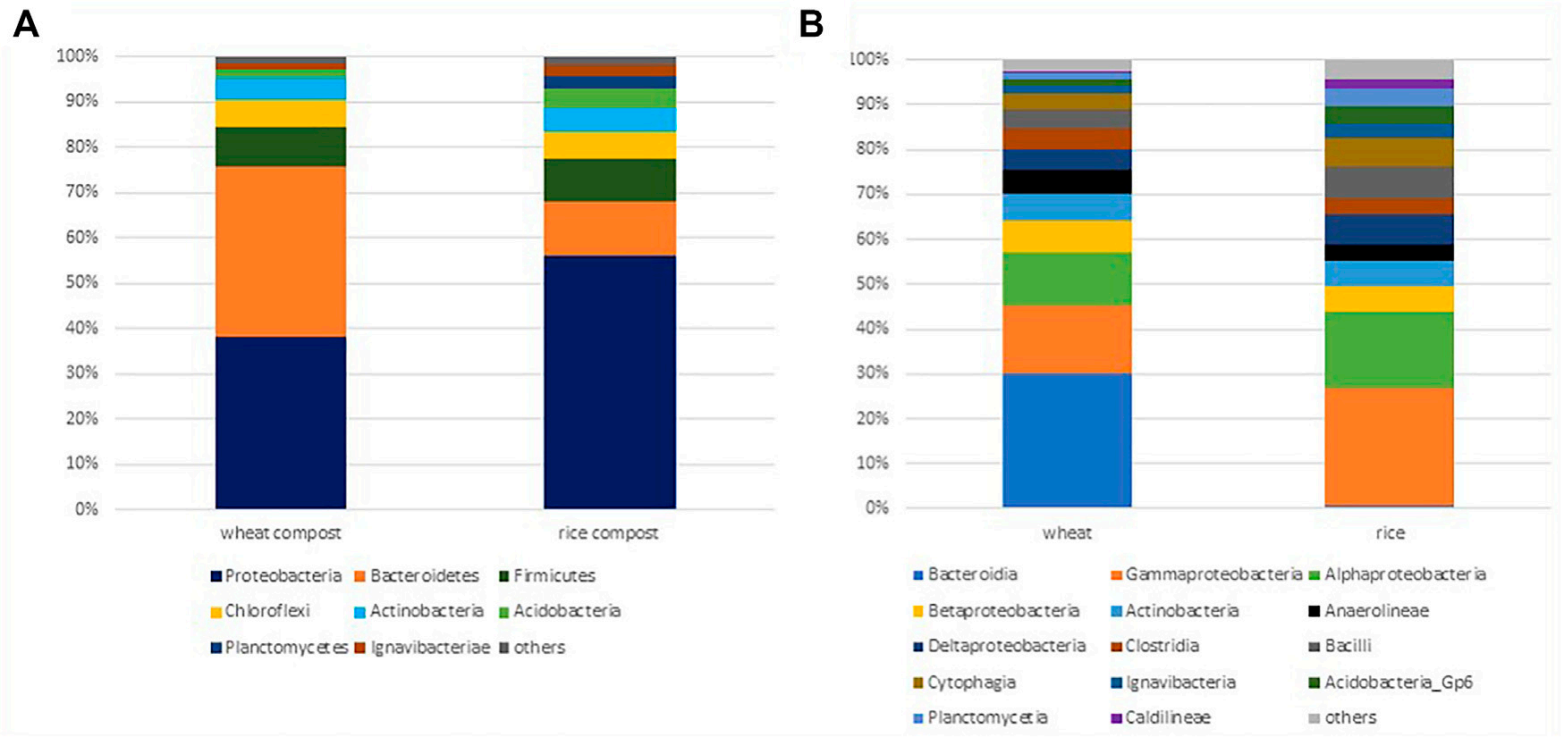
**(A)** Phylum level. **(B)** Class level relative abundance of major bacteria (represented by >0.5% sequences) detected by 16S rRNA gene analysis in wheat and rice compost samples.

The relative distribution of bacterial phyla in the wheat compost and rice compost samples was evaluated using Illumina sequencing of the 16S rRNA gene (Figure 1). About 26,064 high-quality sequences were retrieved from wheat compost, while 12,888 sequences were recovered from rice compost. Sequence analyses revealed the presence of twenty kingdoms/phylum-level groups in wheat compost samples, of which *Proteobacteria* and *Bacteroidetes* were dominant phyla comprising 19.83 and 19.78% sequences, respectively, followed by *Firmicutes* (4.62%), *Chloroflexi* (2.92%), *Actinobacteria* (2.57%), and *Acidobacteria* (1.01%). Twenty phyla accounted for the sequences in the rice compost with eighteen phyla shared with wheat compost samples, while *Pacearchaeota*- and *Spirochetes-*related sequences were only detected in the rice compost. The *Proteobacteria* was the only dominant phylum accounting for 24.58% of the sequences in rice compost followed by *Bacteroidetes* (5.12%), *Firmicutes* (4.22%), *Chloroflexi* (2.57%), *Actinobacteria* (2.29%), and *Acidobacteria* (1.83%).

Taxonomic hit distribution at the class level shows that the class *Bacteroidia* was dominant in the wheat compost comprising 11.48% of sequences, followed by *Gammaproteobacteria* (5.88%) and *Alphaproteobacteria* (4.39%). In rice compost samples, the sequences representing the *Gammaproteobacteria* were by far the largest group accounting for 9.15% of sequences followed by *Alphaproteobacteria* (5.94%). The class *Bacteroidia* of phylum *Bacteroidetes* was underrepresented in the rice compost comprising only 0.19% of sequences, while the classes *Cytophagia* (2.23%) and *Sphingobacteriia* (0.33%) were relatively abundant. The classes *Actinobacteria*, *Anaerolineae*, *Betaproteobacteria*, *Deltaproteobacteria*, *Clostridia*, and *Bacilli* have a similar distribution in both wheat and rice composts comprising 1–3% of total sequences. Most bacterial classes observed were found in both composts, that is, wheat and rice compost, although the difference in the distribution of bacterial sequences was observed.

### 3.2 Appearance and Quality Analysis

The compost color was light brown to dark brown in appearance. Both simple as well as bioactive/enriched compost were ground down and passed through a 2.0-mm sieve to ensure the homogeneity of the product. Characteristic comparison between both types of compost for pH, electrical conductivity (ECe), percentage nitrogen (N) content, phosphorus (P) content, organic matter (OM), and moisture content is given in Table 1, which shows enriched compost has slightly higher pH, ECe but is according to the standard than that of the simple compost. Nitrogen, phosphorus organic matter, water holding capacity, and moisture levels are according to the standard values for the compost. Moreover, a non-significant amount of heavy metals Cu, Pb, Hg, Se, and Cd was detected in both types of composts.

**TABLE 1:** Characteristic of the compost and bioactive compost.

Characteristic	Standard	Compost	Bioactive compost
Organic matter (%)	>20	50–60	50–60
pH	6–8.5	6.9–7.5	7.0–8.0
ECe	4.1	3.3–3.5	3.1–3.4
Total nitrogen (%)	≈2	2.3–3.0	2.7.0–3.1
Total phosphorus (%)	≈2	1.8–1.9	1.8–2.0
*E. coli*	<1,000 MPN/g	0	0
*Salmonella*	<3–4 MPN/g	0	0
Phytotoxicity (seed germination) assay	80–90%	85%	95%
Water holding capacity (g) water/g compost)	4	15.54	20.5
Cu (ppm)	13–20	16.65	17.41
Pb (ppm)		0.7–0.9	0.7–0.9
Hg (ppm)		0.4–0.5	0.4–0.5
Se, Cd (ppm)		0	0
Moisture contents (%)	<70	20	25

### 3.3 Efficiency and Effectiveness of Bioactive Compost

The total viable bacteria cell count of wild strains in simple compost ranged from 3.88 to 5.73 at 7 and 180 dpi, respectively. Total viable cell counts in the compost inoculated with wild PGPR strains continuously increased from 7.0 dpi (6.99) to 90 = dpi (9.57) and decreased at later stages, that is, 180 dpi (7.61). Specific detection of antibiotic-resistant strains showed a similar trend, that is, a continuous increase from 7.0 dpi (6.18) to 90 dpi (9.55) and reduced at the final stage, that is, 180 dpi (7.92) (Table 2). This clearly shows that inoculated PGPR strains survived better in the compost up to 180 dpi.

**TABLE 2:** Total bacterial population analysis on compost and bioactive compost.

Days post-inoculation (dpi)	Log values of viable cells		
	Compost	Compost + wild PGPR strains	Compost + antibiotic-resistant PGPR strains
**7**	3.88 ± 0.65	6.99 ± 0.7	6.18 ± 0.6
15	4.60 ± 1.0	7.29 ± 0.6	7.24 ± 1.2
30	4.47 ± 1.4	8.63 ± 4.5	8.33 ± 2.4
60	4.65 ± 0.7	8.51 ± 5.2	8.53 ± 0.5
90	4.93 ± 4.0	9.57 ± 0.5	9.55 ± 0.6
180	5.73 ± 1.3	7.61 ± 0.5	7.92 ± 0.6

The functional viability and efficiency of PGPR recovered from bioactive compost were repeatedly confirmed at each interval. No change in the P-solubilization ability of WP-2 was observed and remained comparable to that of the pure strain till 180 days. Indole-3-acetic acid production by ER-20, K-1, and WP-2 was also comparable to the pure strain, and no significant change was observed throughout (180 days). Similarly, the nitrogen-fixing ability of K-1 and ER-20 on the nitrogen-free malate (NFM) semisolid medium remained unchanged till 180 dpi.

#### 3.3.1 Analysis of Bacterial Survival

##### FESM and FISH Analysis

**FIGURE 2:**
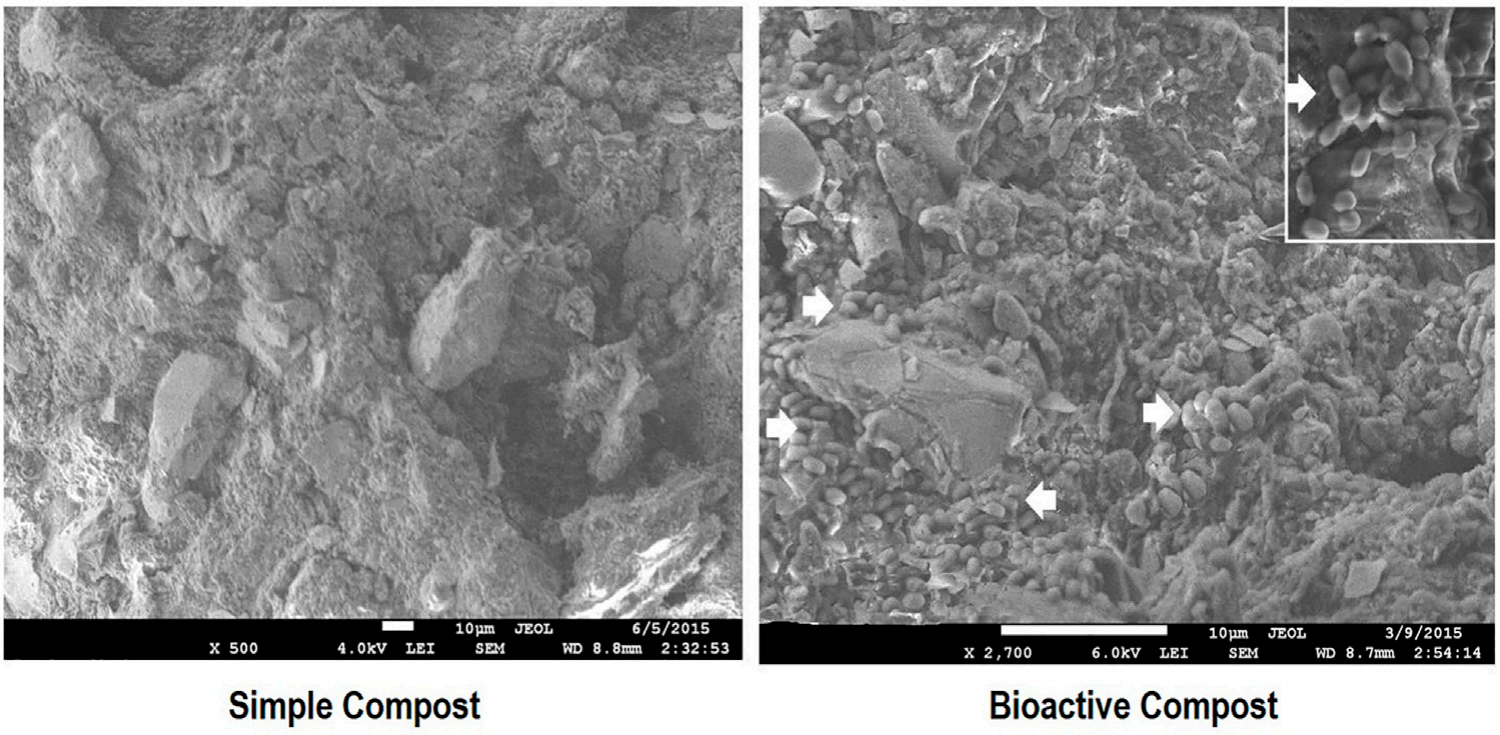
Field emission scanning electron microscopic (FESEM) analysis of inoculated compost at 90 dpi to visualize PGPR composition and compost structure compared to non-inoculated.

FESEM analysis of simple and bioactive compost samples at 30, 60, 90, and 120 dpi showed variations in the morphology of compost in terms of particle size and structure. In bioactive compost, presence and distribution of inoculated PGPR can be seen in a scattered form on the surface as well as in the form of micro-colonies in the grooves of compost that provide micro-niche to the inoculated PGPR (Figure 2). It is clear from FESEM image analysis that the morphology of bioactive compost is better than that of the simple compost. Most probably, high activity and competition of microbes for space and nutrients lead to the creation of grooves and fine-sized compost particles which can accommodate a large microbial population.

**FIGURE 3:**
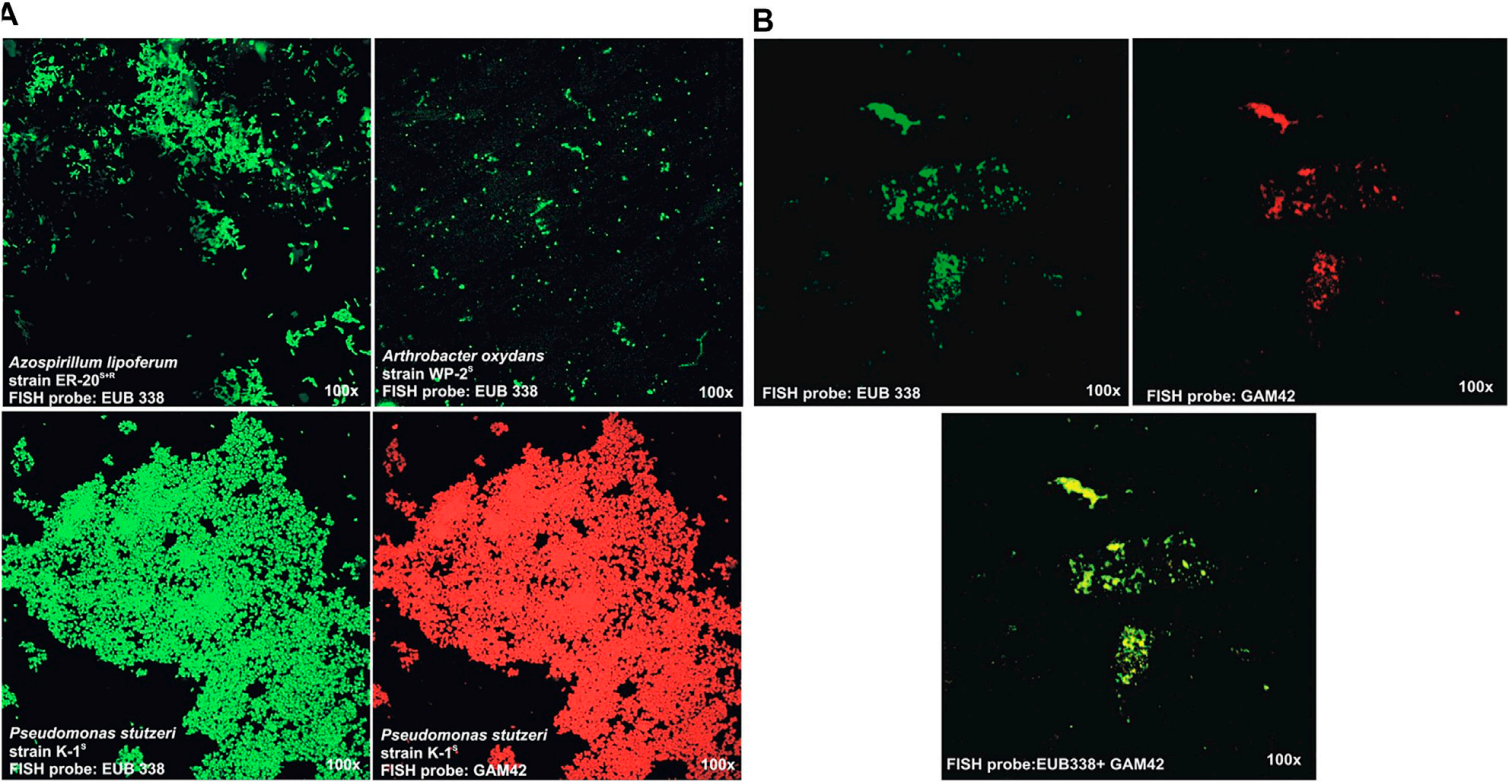
Confocal laser scanning microscopy (CLSM) images of hybridized of *A. brasilense* strain ER-20^S^, *P. stutzeri* strain K-1^S^, and *A. oxydans* strain WP-2^S+R^ labeled with FISH probes (EUB 338-green, GAM42-red): **(A)** pure cultures and **(B)** bioactive compost at 180 dpi

Confocal laser scanning microscopy (CLSM) images of hybridized antibiotic-resistant derivatives pure cultures: *A. brasilense* strain ER-20^S^, *P. stutzeri* strain K-1^S^, and *A. oxydans* WP-2^S+R^ labeled with FISH probes (EUB 338-green, GAM42-red) in Figure 3A show that all the fixation and hybridization conditions were optimum, and the microscope setting was fine to give the fluorescent image of the inoculated bacteria. The CLSM analysis of compost samples processed for FISH captured a population of bacteria at 180 dpi (Figure 3B). However, this technique was able to identify the whole bacterial population at a particular time (using EUB338 probe), as well as Gamma Proteobacteria (using GAM42 probe) population captured on compost seems very low as compared to that which was calculated using viable cell counts after dilution plating. This might be due to the reason that the soil FISH protocol was modified for the fixation and hybridization of the compost samples which needs further optimization.

##### PCR-Based Detection

The DNA was extracted from compost samples in triplicates at 90 and 180 dpi for the detection of strain K-1. The result of PCR showed that *P. stutzeri* strain K-1 survived in the inoculated compost sample after 90 dpi as well as at 180 dpi (Supplementary Figure S1). The presence of 0.9 Kb DNA band in the inoculated compost DNA exactly corresponds to the amplified PCR product in pure culture of *P. stutzeri* strain K-1, which shows the specificity of primers for the detection of *P. stutzeri* strain K-1. Furthermore, the absence of any band in the non-inoculated compost samples showed the absence of any cross-reacting strain or bacterial species having a similar *rrs* sequence with bacterial *P. stutzeri* strain K-1. In compost samples, the PCR product of 0.9 Kb was detected in dilution from 10^−4^ to 10^−6^ at 90 dpi, while at 180 dpi the product was detected in dilution from 10^−2^ to 10^−7^. The absence of any PCR product in lower dilutions and stock DNA may be due to the high humic acid contents of the compost which may hinder the PCR reaction.

### 3.4 Plant Evaluation of Bioactive Compost in Pots

**FIGURE 4:**
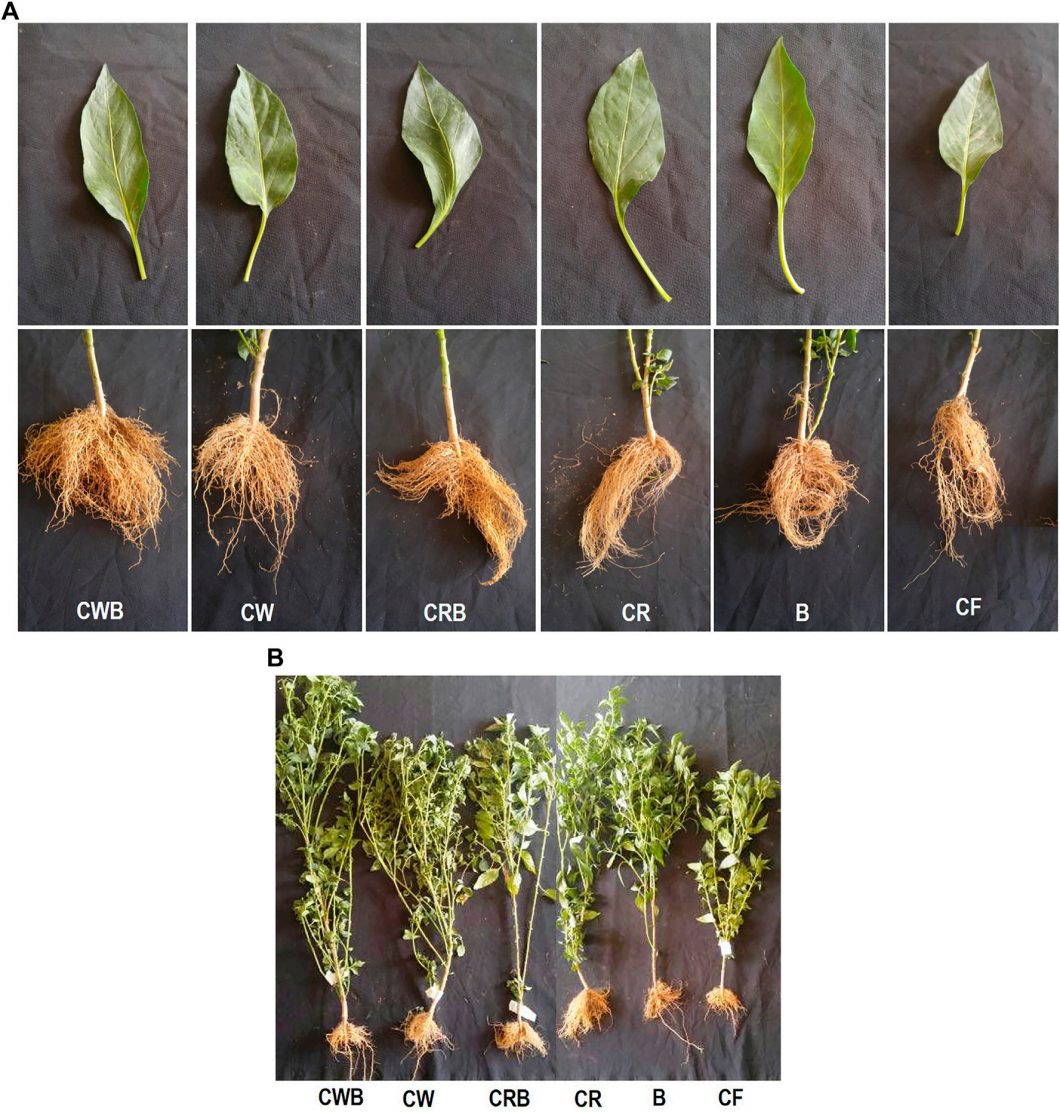
**(A)** Inoculation response of rice and wheat compost and PGPR inoculum on the leaf and root growth of chilies compared with those grown in soil with chemical fertilizers. **(B)** Response of rice and wheat compost and PGPR inoculum on chilies’ growth in pots compared with chili grown in soil with chemical fertilizers.

The PGPR supplementation of compost exerted a significant positive effect on shoot length, shoot fresh weight, and dry weight of chilies in all the inoculated treatments as compared to non-inoculated compost or soil. The shoot growth, root growth, as well as leaf size were significantly better in the compost treatments and PGPR-supplemented treatments where the plants were healthy and strong visually (Figures 4 A,B). Similarly, PGPR stimulated root growth in inoculated treatments (Figure 4A) and shoot growth even when applied in soil (Table 3), which validates the *in vivo* efficacy of the PGPR inoculum. The initial analysis shows that both types of composts have a stimulatory effect on chilies’ growth compared to soil (Figures 4 A,B; Table 3). The root length, shoot length, and plant fresh and dry weights were significantly higher in supplemented compost than in other treatments. On average, the response of the wheat and rice composts with PGPR supplementation was statistically similar. Initially, both wheat and rice composts were tested separately to see the individual impact, but later on, both of these composts were mixed in a 1:1 ratio for further analysis and testing in pots or the microplots.

**TABLE 3:** Individual impact of wheat and rice compost with/without PGPR consortium on the morphological growth of chili in pots.

Treatments	Root length (cm)	Shoot length (cm)	Plant fresh weight (g)	Plant dry weight (g)
Soil + Fertilizer (CF)	15.33 ± 0.21c	43.90 ± 0.66e	34.95 ± 0.43d	7.01 ± 0.29d
Soil + PGPR (B)	17.80 ± 0.36b	49.60 ± 0.10d	40.11 ± 0.39c	9.54 ± 0.29c
W-compost (CW)	17.83 ± 0.45b	50.90 ± 0.46c	44.41 ± 0.89b	11.02 ± 0.17b
W-compost + PGPR (CWB)	20.17 ± 0.35a	55.30 ± 0.36b	49.48 ± 0.68a	13.60 ± 0.35a
R-compost (CR)	17.83 ± 0.49b	50.17 ± 0.35cd	45.64 ± 0.87b	11.11 ± 0.29b
R-compost + PGPR (CRB)	20.47 ± 0.40a	56.13 ± 0.40a	50.64 ± 1.05a	13.58 ± 0.55a
LSD (*p* < 0.05)	0.32	0.34	0.62	0.28

**FIGURE 5:**
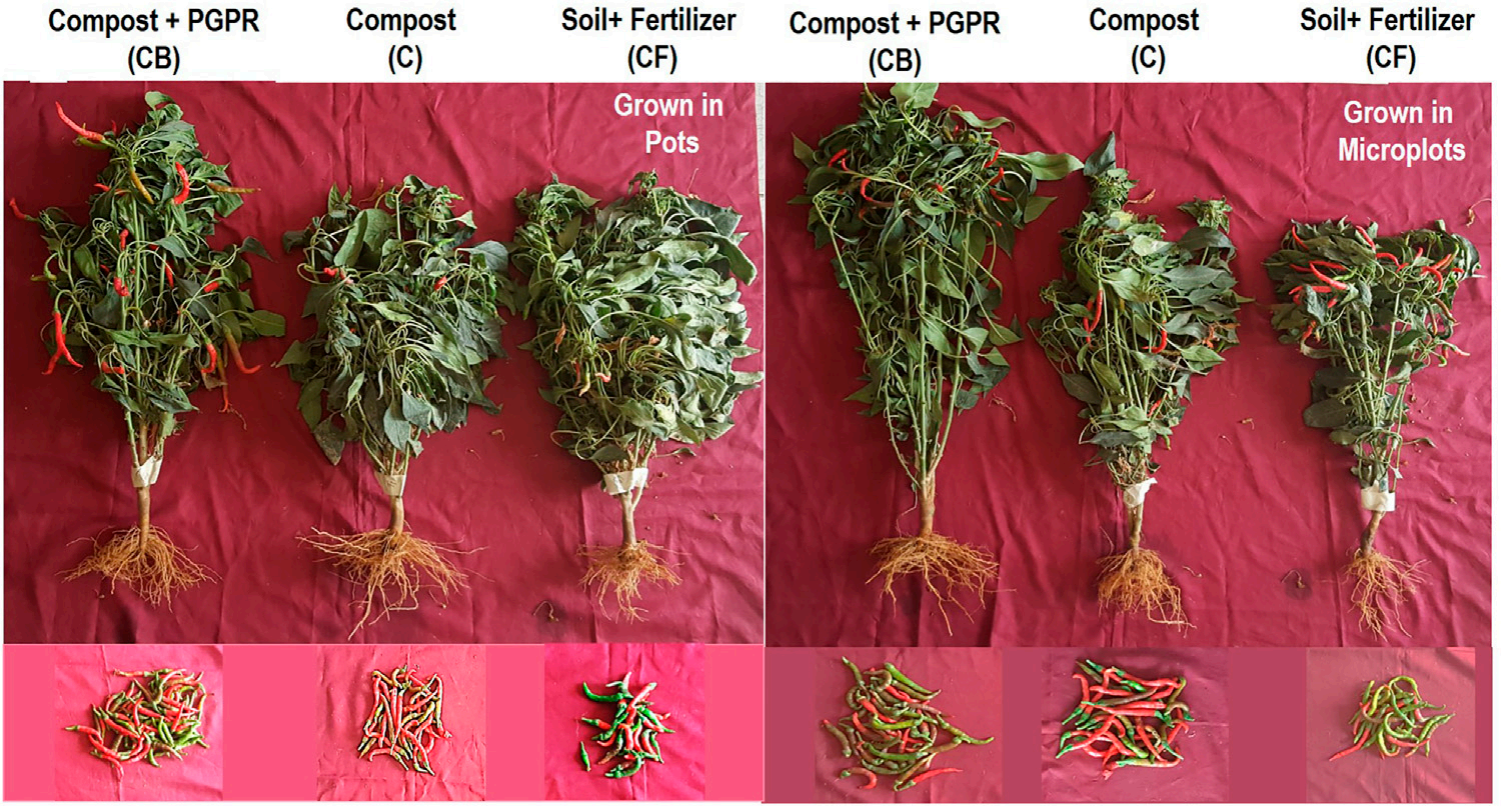
Response of compost and PGPR inoculum on chilies growth and total yield per plant in pots and microplots compared with that grown in soil with chemical fertilizers.

The second experiment where the rice and wheat composts were mixed in a 1:1 ratio shows an increased growth of plant after the addition of compost and PGPR to the root zone (Figure 5A; Table 4). A significant increase in morphological, agronomic, physiological parameters and yield of chilies was observed by supplementation of compost with PGPR as compared to that without PGPR or soil (Table 4). The treatment response was maximum with CB followed by C and CF for all agronomic, physiological, and yield parameters. The addition of bacteria increased the no. of chili and total yield per plant (31.67, 154.02 g) compared to the compost without PGPR (22.00, 98.66 g) and soil (14, 47.55 g). These results validated our hypothesis that PGPR could survive in compost, and this PGPR-supplemented organic formulation exerts stimulatory effects on the growth of chilies.

**TABLE 4:** The combined impact of wheat and rice composts (mixed as 1:1) with/without PGPR consortium on chili growth and yield in pots.

Treatments	Root length (cm)	Shoot length (cm)	Fresh weight/plant (g)	Dry weight/plant (g)
Soil + CF	13.13 ± 0.40c	44.70 ± 1.02c	37.32 ± 0.80c	7.93 ± 0.51c
Compost (C)	17.47 ± 0.31b	55.46 ± 0.83b	46.68 ± 0.76b	12.77 ± 0.54b
Compost + Bacteria (CB)	21.47 ± 0.21a	60.34 ± 0.53a	53.68 ± 0.88a	17.24 ± 0.41a
LSD (*p* < 0.05)	0.26	0.67	0.66	0.40
**Treatments**	**Chilies’ fresh weight (g)**	**Chilies’ dry weight (g)**	**No. of chilies/plant**	**Total yield/plant (g)**
Soil + CF	3.40 ± 0.13c	0.56 ± 0.03c	14.00 ± 1.00c	47.55 ± 3.81c
Compost (C)	4.49 ± 0.18b	0.82 ± 0.04b	22.00 ± 1.00b	98.66 ± 4.87b
Compost + Bacteria (CB)	5.70 ± 0.15a	1.19 ± 0.04a	31.67 ± 1.53a	154.02 ± 4.08a
LSD (*p* < 0.05)	0.13	0.03	0.98	3.49

Testing of the treatments in the microplots showed a similar growth-stimulatory response of compost + PGPR (Figure 5B; Table 5). Maximum plant growth was observed in CB with root length: 18.9 cm, shoot length: 57.6 cm, fresh wt.: 55.7 g, and dry weight: 13.92 g followed by the plants grown in simple compost where root length: 15 cm, shoot length: 52.6 cm, fresh wt.: 46.39 g, and dry weight: 11 g were observed (Table 5). Similarly, the maximum chilies per plant 30, fresh wt. 4.84 g, and dry wt. 1.04 g were observed in plants grown in the compost with PGPR (Table 5). Generally, PGPR-enriched compost showed a percent increase of 19, 40.5% in root length, 8.7, 20% in shoot length, 16.7, 34% in shoot fresh weight, 21, 47% in dry weight, 36.7–56.7% in the number of chilies per plant, 14–24% in average chili fresh weight, 30–52% in chili biomass, and 46–67% in total chili yield (g) over simple compost and soil, respectively.

**TABLE 5:** Effect of PGPR-enriched compost on the growth of chilies grown in microplots.

Treatments	Root length (cm)	Shoot length (cm)	Fresh weight/plant (g)	Dry weight/plant (g)
Soil + CF	11.27 ± 0.35c	45.93 ± 0.42c	36.61 ± 0.72c	7.39 ± 0.24c
Compost (C)	15.30 ± 0.40b	52.60 ± 0.50b	46.39 ± 0.98b	11.01 ± 0.29b
Compost + Bacteria (CB)	18.93 ± 0.25a	57.63 ± 0.21a	55.67 ± 0.96a	13.92 ± 0.29a
LSD (p < 0.05)	0.28	0.32	0.73	0.22
**Treatments**	**Chilies’ fresh weight (g)**	**Chilies’ dry weight (g)**	**No of chilies/plant**	**Total yield/plant (g)**
Soil + CF	3.69 ± 0.20c	0.50 ± 0.04c	13.00 ± 1.00c	48.04 ± 5.91c
Compost (C)	4.16 ± 0.14b	0.73 ± 0.03b	19.00 ± 1.00b	78.95 ± 1.55b
Compost + Bacteria (CB)	4.84 ± 0.15a	1.04 ± 0.04a	30.00 ± 1.00a	145.18 ± 5.86a
LSD (*p* < 0.05)	0.14	0.03	0.82	3.99

#### 3.4.1 Plant Evaluation of Bioactive Compost in Microplots

**FIGURE 6:**
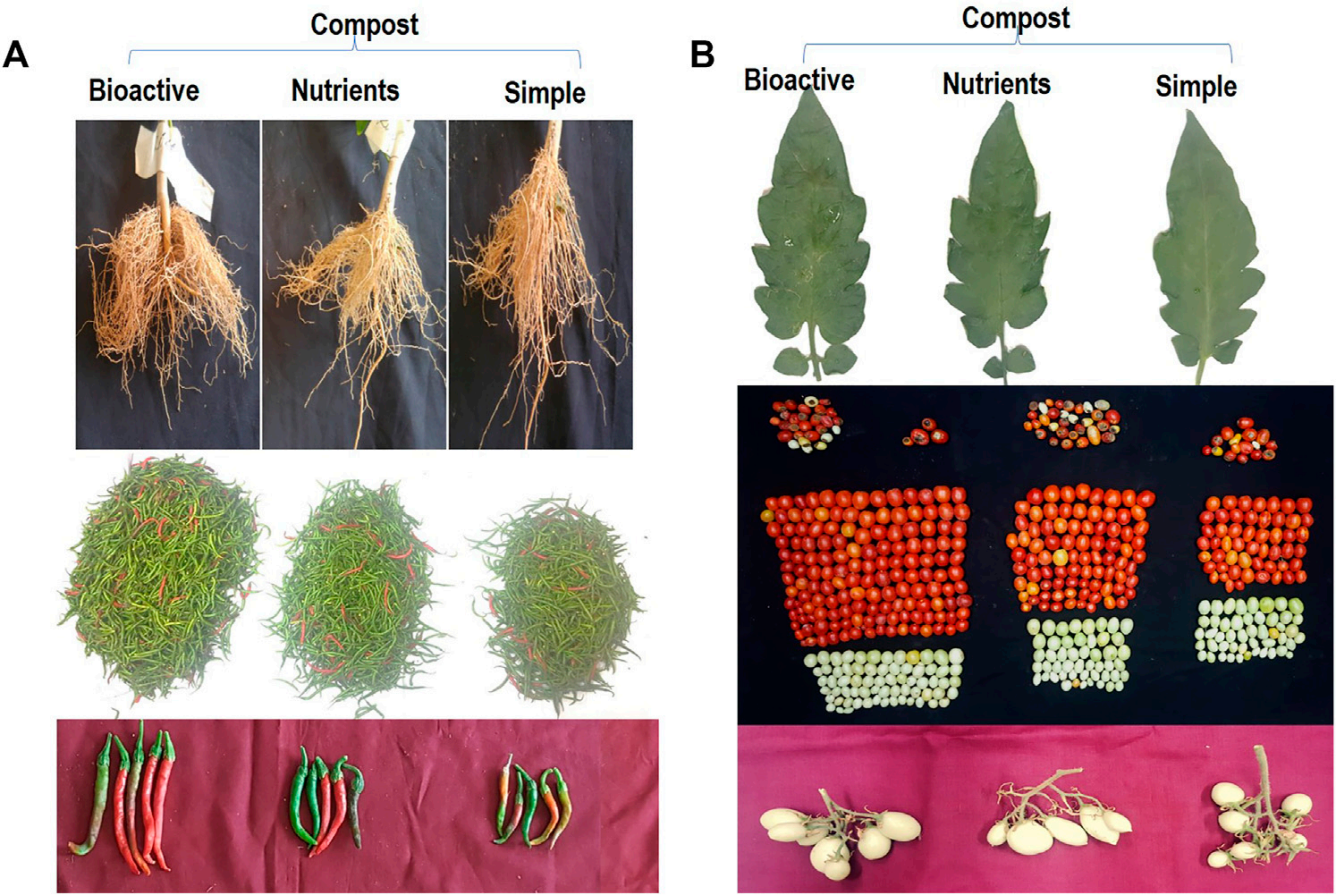
Response of bioactive compost on chilies’ root growth, total yield per plot, and size of the chili **(A)**, and tomato leaf growth, yield per plot (one picking), and the inflorescence **(B)** in microplots compared with other treatments.

Bioactive compost displayed a positive effect on the growth and yield of chili pepper (Figure 6A; Table 6). The root growth, the number of chilies per plot, and the size of the chili were significantly better in the bioactive compost treatment than in other treatments (Figure 6A). Data show that bioactive compost improved chili pepper growth by a 12–22% increase in shoot length, 6–15% in plant fresh weight, and 18–26% in plant fresh dry weight as compared to that of the nutrient-enriched compost and simple compost, respectively, while nutrient-enriched compost showed a 21% increase in root length than that of the bioactive compost. But again, the augmented effect of bioactive compost was observed in different yield-related parameters like 34–46% increase in the number of chilies per plant and the total number of chilies and similarly 66–75% in the fresh weight of chilies per plant and overall fresh weight of total yield of chilies. Bioactive compost also increased 11–19% nitrogen content and 12–19% phosphorus content of chilies as compared to that of the nutrient-enriched compost and simple compost, respectively. Leaf photosynthetic parameters showed variable responses to different treatments; diffusive resistance was 94%, and relative humidity was higher in the simple compost than in the bioactive compost. Leaf temperature was more or less similar in all treatments, but 18–31% increase in quantum and 25–41% in the transpiration rate showed by bioactive compost compared to the other two treatments, respectively. Significant increase in chlorophyll pigments (a, 22–35% + b, 16–37%) 20–35%, and 12–28% in carotenoid contents were observed. Similarly, 11–36% increase in soil phosphatase and 18–46% in dehydrogenase with bioactive compost as compared to that of the nutrient-enriched compost and simple compost, respectively (Table 6), were observed.

**TABLE 6:** Effect of bioactive compost on growth parameters of chilies grown in small tunnel for 2 years (2 years averaged data).

Parameters		Treatments			LSD (*p* < 0.05)
		Simple compost (C)	Nutrient-enriched compost (CN)	Bioactive compost (BAC)	
Plant growth parameters	Root length (cm)	10.83 ± 0.25c	13.87 ± 0.45b	16.80 ± 0.30a	0.28
	Shoot length (cm)	43.10 ± 1.51c	55.73 ± 1.60b	63.43 ± 1.65a	1.30
	Plant FW (g)	156.80 ± 2.55c	172.83 ± 6.94b	185.37 ± 6.38a	4.61
	Plant DW (g)	39.90 ± 1.21c	44.50 ± 0.96b	54.07 ± 1.56a	1.04
Plant yield parameters	No of chilies/plant	85.47 ± 4.52c	105.13 ± 2.07b	159.33 ± 3.42a	1.27
	Yield of chilies/plant (g)	266.66 ± 14.1c	363.76 ± 7.15b	1,058.6 ± 21.36a	5.60
	Total no of chilies	1,280.7 ± 14.15c	1,576.3 ± 13.80b	2,390.5 ± 44.6a	10.28
	Total yield of chilies (Kg)	3.999 ± 0.445c	5.4541 ± 0.477b	16.102 ± 6.54a	13.79
	Total N in fruit (mg/g)	0.184 ± 0.004c	0.206 ± 0.004b	0.226 ± 0.004a	0.004
	Total P in fruit (mg/g)	0.211 ± 0.004c	0.229 ± 0.004b	0.259 ± 0.003a	0.003
Photosynthetic activity	Diffusive resistance (s/cm)	2.20 ± 0.05a	1.50 ± 0.05b	1.13 ± 0.029c	0.04
	Quantum (µmol/sm^2^)	753.5 ± 36.4c	895.9 ± 22.2b	1,096.5 ± 27.5a	23.9
	Transpiration rate (µg/Scm^2^)	17.37 ± 1.4c	22.13 ± 1.5b	29.37 ± 1.43a	1.17
	Relative humidity (%)	28.00 ± 0.5a	27.67 ± 0.8a	27.50 ± 0.5a	0.49
	Leaf temp.(°C)	36.867 ± 0.4a	37.07 ± 0.3a	37.03 ± 0.42a	0.31
Photosynthetic pigments	Chlorophyll a (mg/gFW)	0.57 ± 0.030c	0.68 ± 0.023b	0.87 ± 0.056a	0.032
	Chlorophyll b (mg/gFW)	0.27 ± 0.027c	0.36 ± 0.03b	0.43 ± 0.026a	0.023
	Total Chl. (mg/gFW)	0.84 ± 0.052c	1.03 ± 0.05b	1.29 ± 0.072a	0.048
	Carotenoids (mg/gFW)	0.424 ± 0.015c	0.52 ± 0.01b	0.59 ± 0.010a	0.0097
	Chl. a/b ratio	2.11 ± 0.153a	1.90 ± 0.11a	2.04 ± 0.132a	0.1084
Soil enzyme	Phosphatase EU (10^2^) µg/ml	6.05 ± 0.22c	8.45 ± 0.33b	9.45 ± 0.30a	0.1298
	Dehydrogenase EU (10^2^) µg/ml	3.45 ± 0.11c	4.74 ± 0.19b	5.78 ± 0.17a	0.2325

Bioactive compost also displayed a positive effect on the visual growth and yield of tomatoes (Figure 6B). Leaf photosynthetic parameters and pigment contents were also improved by the treatment with bioactive compost and nutrient-enriched compost as compared to the application of compost. Bioactive compost displayed a 7–13% increase in plant height, 7–22% in stem diameter, 12–24% in leaf area, 18–28% in the number of lateral branches per plant, and 9–21% in their length. Similarly, in yield parameters, for example, 26–44% increase in fruit fresh weight, 35–55% in fruit dry weight, 22–39% in fruit diameter, 27–56% in the number of marketable fruits per plant and the total number of marketable fruits, 46–75% fresh weight of marketable fruits and total fresh weight of marketable fruits as compared to that of the nutrient-enriched compost and simple compost, respectively, were observed. But approximately 45% increase and 4% decrease in the number of non-marketable fruits per plant, total non-marketable fruits, fresh weight of non-marketable fruits, and total fresh weight of non-marketable fruits were also observed in bioactive compost–treated plants as compared to simple compost and nutrient-enriched compost, respectively. Fruits of bioactive treated plants were 13–25% richer with total nitrogen and 13–19% with phosphorus contents. Furthermore, leaf photosynthetic parameter response was mixed. All the treatments were non-significant for quantum and leaf temperature. Like chilies, diffusive resistance was also 66% higher in simple compost–treated plants than bioactive compost treatment, and non-significant with nutrient-enriched compost. Similarly, the transpiration rate and relative humidity treatment with simple compost and nutrient-enriched compost were non-significant, but bioactive compost showed 43–47% and 10–12% increase, respectively. Bioactive compost also showed a 20–31% increase in chlorophyll a, 38–49% in chlorophyll b, and a total of 26–37% in chlorophyll (a + b) and 11–21% in carotenoid contents as compared to that of nutrient-enriched and simple compost, respectively (Table 7).

**TABLE 7:** Effect of bioactive compost on growth, yield, physiological and photosynthetic pigments of tomatoes grown in small tunnel, and post-harvest activity analysis of enzymes in soil (2 years averaged data).

Parameters		Treatments			LSD (*p* < 0.05)
		Simple compost (C)	Nutrient-enriched compost (CN)	Bioactive compost (BAC)	
Plant growth parameters	Plant height (cm)	132.390 ± 3.758b	140.767 ± 4.128b	151.597 ± 4.804a	3.4718
	Stem diameter (cm)	1.150 ± 0.050c	1.367 ± 0.029b	1.467 ± 0.029a	0.0304
	Leaf area	31.920 ± 1.502c	36.857 ± 1.925b	41.857 ± 2.804a	1.7527
	No. of lat. branches/plant	22.500 ± 0.901c	25.567 ± 0.957b	31.233 ± 1.776a	1.0418
	Lateral branch len. (cm)	95.35 ± 4.927c	110.033 ± 3.956b	120.250 ± 5.212a	3.8610
Plant yield parameters	Fruit fresh weight (g)	43.63 ± 2.38c	57.89 ± 1.99b	77.81 ± 2.49a	1.8756
	Fruit dry weight (g)	5.94 ± 0.079c	8.53 ± 0.45b	13.15 ± 0.26a	0.2468
	Fruit diameter (cm)	3.12 ± 0.1c	3.98 ± 0.15b	5.12 ± 0.17a	0.1161
	No. of marketable fruits/plant	49.67 ± 3.51c	82.33 ± 4.51b	112.58 ± 8.16a	4.6949
	Marketable yield/plant (g)	2,172.69 ± 270.62c	4,764.05 ± 245.05b	8,766.4 ± 795.2a	412.49
	Total no. marketable fruits	298 ± 21.071c	494 ± 27.06b	675.5 ± 48.94a	28.169
	Total marketable yield(g)	13.04 ± 1.62c	28.58 ± 1.47b	52.6 ± 4.77a	2.4750
	No. of non-marketable fruits/plant	28 ± 3.61a	29 ± 3a	15.33 ± 1.53b	2.3254
	Non-marketable yield/plant (g)	140 ± 18.03a	145 ± 15a	76.67 ± 7.64b	11.627
	Total non-marketable yield(g)	92 ± 9.17b	174 ± 18a	168 ± 21.63a	13.952
	Total non-marketable yield(g)	0.46 ± 0.05b	0.87 ± 0.09a	0.84 ± 0.11a	0.0698
	Total N in fruit (mg/g)	0.165 ± 0.003c	0.19 ± 0.002b	0.219 ± 0.002a	0.0021
	Total P in fruit (mg/g)	0.259 ± 0.003c	0.276 ± 0.004b	0.318 ± 0.005a	0.0032
Physiological parameters	Diff. resistance (s/cm)	3.040 ± 0.132a	3.003 ± 0.153a	1.830 ± 0.053b	0.0986
	Quantum (µmol/sm^2^)	646.047 ± 17.252a	655.507 ± 10.410a	658.110 ± 15.985a	12.125
	Trans. rate (µg/Scm^2^)	12.903 ± 0.202b	12.027 ± 0.175b	22.603 ± 0.734a	0.3680
	Relative humidity (%)	11.697 ± 0.230b	11.443 ± 0.454b	12.987 ± 0.414a	0.3090
	Leaf temp. (°C)	36.393 ± 0.614b	36.877 ± 0.866 ab	37.993 ± 0.340a	0.5254
Photosynthetic pigments	Chlorophyll a (mg/gFW)	0.687 ± 0.012c	0.794 ± 0.007b	0.994 ± 0.022a	0.0123
	Chlorophyll b (mg/gFW)	0.234 ± 0.007c	0.284 ± 0.005b	0.457 ± 0.027a	0.0131
	Total Chl. (mg/gFW)	0.921 ± 0.012c	1.077 ± 0.002b	1.450 ± 0.005a	0.0062
	Carotenoids (mg/gFW)	0.470 ± 0.010c	0.527 ± 0.006b	0.593 ± 0.006a	0.0061
	Chl. a/b ratio	2.940 ± 0.115a	2.799 ± 0.073a	2.182 ± 0.176b	0.1046
Soil enzyme activity	Phosphatase EU (10^2^) µg/ml	6.970 ± 0.255c	9.107 ± 0.215b	10.953 ± 0.195a	0.1822
	Dehydrogenase EU (10^2^) µg/ml	4.487 ± 0.180c	5.603 ± 0.130b	6.593 ± 0.185a	0.3335

##### Fruit Nutrient Analysis

Total N and P estimation in tomato and chili fruits showed a significantly higher amount of NP in the fruits. Chilies treated with the bioactive compost showed an 18.7 and 18.5% increase in NP, respectively, in comparison to the simple compost, while 9 and 11.7% increase in NP, respectively, in comparison to the nutrient-enriched compost. Similarly, bioactive compost–treated tomatoes showed a 24.7 and 18.5% increase in NP contents than the simple compost and approximately 13.2% than the nutrient-enriched compost (Tables 6, 7).

##### Effect on Soil Enzymatic Activity

In the chilies’ and tomatoes’ microplot experiments, both alkaline phosphatase activity and dehydrogenase activity of soil treated with bioactive compost were significantly higher than those of the nutrient-enriched or simple compost. In chilies, post-harvest soil analysis showed an increase of 11–36% in alkaline phosphatase and 18–46% in dehydrogenase activity compared with nutrient-enriched compost and simple composts, respectively (Table 6). Similarly, in tomatoes, 17–36% increase in alkaline phosphatase and 15–32% in dehydrogenase activity with bioactive compost as compared to that of nutrient-enriched compost and simple compost, respectively (Table 7).

##### Total Bacterial Population

Analysis of the bacterial population in the rhizosphere from 7 dpi to 90 dpi shows that bacterial count remained the same in the soil, while it increased in the rhizosphere of compost and bioactive compost–treated plants. In soil, bacterial count remained 8.18–8.55, while it was increased from 8.58 to 11.73 and 9.75–12.20 in case of compost and bioactive compost, respectively (Table 8).

**TABLE 8:** Effect of bioactive compost on total viable bacterial population.

Days post-inoculation (dpi)	Log values of viable cells		
	Soil (CF)	Compost (C)	Bioactive compost (BAC)
7	8.18 ± 0.56	8.58 ± 0.65	9.75 ± 0.21
15	8.24 ± 1.27	9.10 ± 1.11	9.92 ± 0.39
30	8.33 ± 2.41	10.07 ± 1.43	10.79 ± 0.94
60	8.53 ± 0.54	11.15 ± 0.74	11.95 ± 2.14
90	8.55 ± 0.56	11.73 ± 1.25	12.20 ± 1.32

## 4 Discussion

Extensive cultivation of staple cereals in response to rising global food demand has brought the consequences in the form of increased agricultural wastes, polluted environment, and nutrient-deprived soils. Moreover, the tunnel farming industry is developing at an exponential rate to fulfill the growing demand for off-season vegetables, fruits, and spices. Tunnel system uses plenty of chemical fertilizers and pesticides; as a result, vegetables/fruits are loaded with chemicals that subsequently cause serious health concerns in human beings. Reliance on chemical fertilizers not only reduces land productivity but also decreases the product quality. The plants grown in this way do not develop good plant characteristics such as good root system, shoot system, and nutritional characters and also will not get time to grow and mature properly. The use of organic fertilizers and biopesticides is highly recommended to improve the quality of soil, and the nutritional value of vegetables and fruits, and to make them suitable for human consumption. This study describes the re-utilization of organic crop waste for the plant fertilization after composting and enrichment with plant-beneficial bacteria.

Wheat and rice straw were co-composted with nitrogen-rich green plants *Sesbania*, *Trifolium*, and *Azolla*. The purpose of co-composting with N-rich green plants was to decrease the C: N ratio and speed up the composting process. Both wheat and rice straw have high lignocellulosic content (high C: N ratio) which hinders the degradation and delays the composting process (Chen, 2014; Snelders et al., 2014). Nitrogen serves as a limiting factor during composting because it is an essential element for the microbe-driven composting process (Rovshandeh et al., 2007). Therefore, the C: N ratio is a good indicator of nitrogen availability as well as microbial activity. It has also been associated with temperature changes in the compost. In a recent study on wheat straw compost, temperature rise was observed with urea supplementation as compared to the non-supplemented compost. Both high microbial activity and temperature help to attain earlier maturity/stability of the compost (Zhang et al., 2016), but high temperature with high pH may lead to nitrogen loss through ammonia production as well (Diaz and Savage, 2007). Analysis of compost metagenome shows that *Proteobacteria* and *Bacteroidetes* were the most abundant phyla in wheat compost comprising 40% of the sequences, whereas in rice compost, *Proteobacteria*, particularly from class Gammaproteobacteria, were dominant. However, the phyla *Firmicutes*, *Chloroflexi*, *Actinobacteria*, and *Acidobacteria* showed a similar distribution in both compost samples accounting for 1–5% of total sequences. The bacteria from γ-*Proteobacteria*, *Firmicutes*, and *Actinobacteria* are reported to be highly involved in the maturation of composting during the final stage of the process, and their relative abundance is reported as an indicator of disease suppression during the process (Hadar and Papadopoulou, 2012). The *Bacteroidetes* are facultative anaerobic bacteria that are considered important during the initial and final stages of composting. Similarly, the higher abundance of *Bacteroidetes*, *Chloroflexi*, and *Planctomycetes* has been reported in mature compost (Kästner and Miltner, 2016).

Several quality factors are considered important while using compost for soil application (Milinković et al., 2019), which include maturity, nutrient content, electrical conductivity, pH, phytotoxic compounds, and contents of pollutants (Sæbø and Ferrini, 2006). The pH range of compost developed in this study was 6.9–7.5 without the addition of urea, while it was 7.3–7.9 with urea-added during composting, which is within the recommended pH range (6.9–8.3) for the compost (Ameen et al., 2016). Slightly higher pH in urea-supplemented compost indicates the production of ammonia (Haddadin et al., 2009) because high temperature and higher pH are associated to cause N-loss through ammonia volatilization (Diaz and Savage, 2007), but the temperature remained almost the same for both heaps (with or without urea) in this study, so there is a possibility that ammonia is condensed back to the compost due to sheet cover. A substantial increase in EC values (3.36–7.5) was observed in both types of composted materials which is in accordance with already reported results for the composting of wheat straw (Zhang et al., 2016). The moisture content of composting material was maintained at 50–55% which is the optimum level to facilitate the decomposition process by providing oxygen for the microbial activity because it is considered as one of the limiting factors for solid substrate (Pace et al., 1995; Jusoh et al., 2013). Both higher and lower moisture contents cause anaerobic conditions that lead to slower decomposition (Sherman, 1999; Diaz and Savage, 2007). In this study, rice and wheat straw of particle size of <2 cm with green plants of particle size >2 cm resulted in efficient degradation and composting of raw material. Studies have reported that particle size has a direct effect on the composting process and the quality of the final product as well. Calabi–Floody et al. (2018) have evaluated three particle sizes <1, 1–2, and >2 cm with 3 nitrogen doses and 3 fungal charges, and concluded that particle size <1 cm with 0.98 g/kg nitrogen dose and the 14-disc fungal charge is optimal for the good quality compost. Since microbes catalyze the transformation so, the higher surface area of the substrate means higher substrate availability for the microbial process (Agnew and Leonard, 2003). In this study, efficient composting was achieved with a relatively larger particle size possibly due to co-composting with the nitrogen-rich green plants and an additional dose of nitrogen (urea) fertilizer. It is a more convenient and practical approach to use a relatively larger particle size straw for large-scale production of compost rather than grinding to a fine size particle which is a labor-intensive and energy-consuming process. The final size of compost was maintained at 2 mm, which has been recently reported to be the best particle size for the final compost product by Tang et al. (2020).

To enhance the organic benefits, the compost was enriched with the plant-beneficial bacteria and micronutrients. Making the compost biologically active multiplies its agricultural and environmental benefits as the organic and biofertilizers work in synergy (Neugart et al., 2018). The plant-beneficial bacteria are highly active in converting the unavailable forms of nutrients (N, P, Zn, *etc*.) and stimulate the root and shoot growth by producing phytohormones. Organic matter helps in the survival of bacteria in the product as well as in the rhizosphere. The better the survival of bacteria in compost, the higher will be the efficacy of the final product (Siddiq et al., 2018). The present study demonstrates the survival and activity of bacteria up to 180 dpi that is due to high organic matter, wide surface area, and optimal water holding capacity. Moreover, it is easily available and inexpensive for mass production. Siddiq et al. (2018) reported compost as good carrier material for PGPR inoculum with higher physiological activity and long shelf life of inoculated PGPR. Sohaib et al. (2020) further supported this hypothesis that an ideal carrier material should have higher organic matter, surface area, moisture content, and neutral pH, and be favorable for the growth of bacteria along with the fact that it should be easily available and inexpensive. Such carrier material provides a selective advantage to PGPR to survive under stressful conditions. Moisture levels 30–50% have been reported as optimal for a carrier material (Sangeetha and Stella, 2012). Wheat straw compost has been already reported as a carrier material for nitrogen-fixing cyanobacteria (Dhar et al., 2007). SEM analysis of compost showed the survival of microbes as dispersed cells on the surface and micro-colonies in the grooves. It further showed efficient degradation of lignocellulose and the development of micro-niche for the microbes. It has been reported that grooves on the surface of carrier material provide a microenvironment to the PGPR for the colonization and better performance of a differential physiological activity (Roy et al., 2010). Scanning electron microscopy (SEM) can effectively monitor the microstructures and grooves (Dresbøll and Magid, 2006), and has been used to study the homogeneity, particle size, pore size, maturity, and stability of rice and wheat straw (Arora and Kaur, 2019; Bhattacharyya et al., 2020) for downstream application as a carrier material. The bacterial presence and survival were further validated using FISH, but the detection limit remained low in this study contrary to that reported previously for compost samples (Franke–Whittle et al., 2005). This might be due to the sensitivity of the probe or the fixation and hybridization conditions used for the compost sample in this study which might affect the detection of the selected population. A specific primer set developed against inoculated *P. stutzeri* strain K-1 (Mirza et al., 2006) was used for the PCR-based detection of K-1 at 90 and 180 dpi which shows that the bacterium in the consortium can survive in the compost. The primer specificity has already been reported on other *Pseudomonas* including non-nitrogen fixing strains and other related species (Mirza et al., 2006).

Application of PGPR with rice and wheat straw compost in this study has significantly improved chili and tomato plants’ fresh and dry weight, leaf photosynthetic efficiency, and overall yield. Several studies have reported that compost (with or without PGPR) improve the growth, yield, and disease tolerance of chili (Ahmad et al., 2011; Kausar et al., 2014; Nur et al., 2019), and increased soil fertility (Rahman et al., 2012). Similarly, the beneficial impact of compost has been documented on tomatoes (Rasool et al., 2021). Compost or vermiculate compost enriched with a multi-strain consortium or potassium humate have shown salt stress mitigation in wheat (Sohaib et al., 2020), seed germination in barley (El-Akshar et al., 2016), and improved sorghum production (Hameeda et al., 2007). PGPR fortified rice straw compost protected rice from blast disease (Ng et al., 2016), and nitrogen-enriched wheat straw compost carrying PGPR improved nutrient use efficiency and seed quality of sunflower (Arif et al., 2017).

Moreover, total N and P contents of fruits were also increased with the application of different nutrient-enriched compost and bioactive compost. It has been well established that to some extent, PGPR and the compost can be used as alternatives to NPK fertilizers because they can enrich the soil with these nutrients (Sarwar et al., 2018; Laslo and Mara, 2019). PGPR colonize the roots and assist the plant in the uptake of NPK (García et al., 2004); that is why a prominent increase in the growth, yield, and nutrient content in tomatoes and chilies was observed with bioactive compost. But the bacterial population and their affinity with the plant roots are itself dependent on several factors including properties of soil, nature of root exudates, and quorum-sensing signaling molecules (Brimecombe et al., 2000; Nawaz et al., 2020). Compost has been also reported to increase the population of beneficial microbes in the soil and rhizosphere (Arancon et al., 2006). Similarly, in this study, total bacterial population in the rhizosphere was increased with the compost compared to that of the soil. This clearly shows that the organic product has supported the bacterial activity in the rhizosphere which was then resulted in the form of better plant growth and yield.

Post-harvest soil enzymatic analysis of tomato and chili rhizosphere soil showed a significant increase in soil alkane phosphatase and dehydrogenase activities. Compost dehydrogenase and alkaline phosphate activity is a measure of microbial activity and maturity (Gaind and Nain, 2010). Soil dehydrogenase activity indicates microbial activity (Salazar et al., 2011), while soil alkaline phosphate activity, on the other hand, is used as a measure of soil richness along with the microbial activity (Versaw and Harrison, 2002). It has been reported previously that the rice straw compost showed a significant increase in soil alkaline phosphatase and dehydrogenase activities with rice yield comparable to the inorganic fertilizer (Goyal et al., 2009). A significant increase in the activity of both enzymes with reduced plant uptake for heavy metals Cd and Zn by rice straw compost has been reported (Tang et al., 2020) mainly due to EC and available potassium content. Long-term application of wheat straw compost enhances soil enzyme activity, reduces the availability of heavy metals Cu and Cd (Xie et al., 2009), and improves the growth of maize, wheat, soybean, pearl millet, and sorghum (Liu et al., 2010).

### 4.1 Cost Benefit Analysis and Policy Recommendations

The cost of tomatoes grown in the tunnel is 56% higher than that of those grown without the tunnel (Khan and Khan, 2020); due to higher inputs (costs of seeds, fertilizers, pesticides, irrigation, and labor), the benefit–cost (B/C) ratio is, however, high, that is, 2.29 for tomatoes grown with tunnel compared to tomatoes grown without tunnel, where the B/C ratio is 1.48. Fertilizers are the major input cost to any crop grown, and changing their prices changes the overall economics of any crop. This study recommends the use of bioactive compost (BAC) without any additional chemical fertilizer, thus further reducing the input cost with a simultaneous increase in yield which further guarantees an even higher B/C ratio for the tunnel system as well as the simple field. In tomato and chili, it is recommended to use 100 Kg BAC per acer of the tunnel and mix before forming beds. It is further recommended that in cereals or other crops which require higher fertilizer inputs, BAC at 100 Kg per acer should be mixed during the field preparation with concomitant decrease in the chemical fertilizers applied during field preparation or seed sowing. This may be followed by the 50% use of the recommended second or third chemical fertilizer dose application as well. The overall outcome will be a lesser chemical input and higher yield returns with low chemical residues in the fruits and seeds.

## 5 Conclusion

Application of enriched organic fertilizers and bioactive compost to infertile agricultural lands could have enormous benefits both environmentally and economically. The present study demonstrates the plant-beneficial impact of bioactive compost and suggests using this bioactive compost in the organic farming of tomatoes and chilies, both in the tunnel and in the field yielding high VCR for both crops. The conversion of farm waste into compost is an effective approach to manage rapidly increasing farms waste. Enrichment of compost with beneficial bacteria will further improve its efficacy and enhance the impact as demonstrated in the present study.

## Data Availability

The datasets presented in this study can be found in online repositories. Compost metagenome data can be retrieved from NCBI database through Bio-Project PRJNA773634 while the 16SrRNA data is available under accession nos. HE662867, HE661626, AJ278107.
