# Supplementary material for: Tailored Bioactive Compost from Agri-Waste Improves the Growth and Yield of Chili Pepper and Tomato
Source: Front Bioeng Biotechnol. 2022 Jan 24;9:787764. doi: 10.3389/fbioe.2021.787764 (PMC8819593; doi:10.3389/fbioe.2021.787764)
Supplement: Supplementary file 1 [file DataSheet1.docx]

Bioactive compost from plant waste improves the growth and yield of chili pepper and tomato

Asma Imran^1*^, Fozia Sardar^¥^, Zebish Khaliq^¥^, Muhammad Shoib Nawaz, Atif Shehzad, Muhammad Ahmad, Sumera Yasmin, Sughra Hakim, Babar S. Mirza, Fathia Mubeen, Muhammad Sajjad Mirza

^1^Institute for Biotechnology and Genetic Engineering (NIBGE), P.O. Box 577, Jhang Road, Faisalabad, Pakistan

^¥^Both authors contributed equally

***Correspondence:**

Asma Imran

asmaaslam2001@yahoo.com


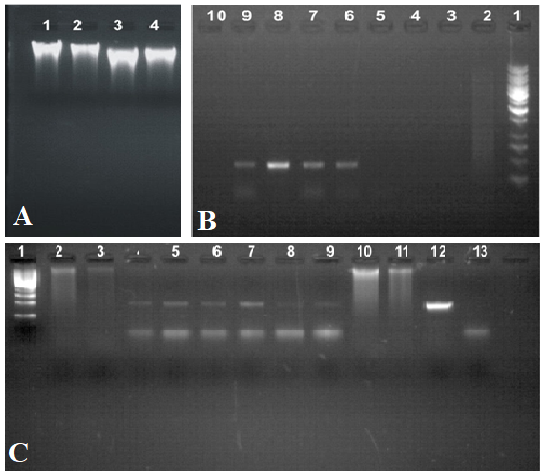


**Supplementary Figure 1|** **Gel picture showing the PCR based detection of** ***P. stutzeri* strain K-1 at 90 dpi from compost**

Lane 1 = 1 Kb DNA marker, Lane 2 = Stock compost DNA, Lane 3-9 = DNA dilution 10-1 to 10-7, Lane 10-11 = Non-inoculated compost (mock) DNA, Lane 12 = P. stutzeri strain K-1S pure culture, Lane 13 = negative control

| Table S1 \| Fluorescently labelled oligonucleotide probes used in FISH analysis of carrier materials | | | | |
| --- | --- | --- | --- | --- |
| Probe name | **Sequence (5´-3´)** | **Specificity** | **Target site** | **Reference** |
| EUB338-I | GCT GCC TCC CGT AGG AGT | Most Bacteria | 16S *rRNA*,  338-355 | ([Amann et al., 1995](#_ENREF_1)) |
| EUB338-II | GCA GCC ACC CGT AGG TGT | Bacteria not covered by probe EUB338-I, e.g., many Planctomycetes | 16S *rRNA*,  338-355 | ([Daims et al., 1999](#_ENREF_2)) |
| EUB338-III | GCT GCC ACC CGT AGG TGT | Bacteria not covered by probe EUB338-I, e.g., many Verrucomicrobia | 16S *rRNA*,  338-355 | ([Daims et al., 1999](#_ENREF_2)) |
| GAM42a | GCCTTCCCACATCGTTT | Gamma Proteobacteria | 23S,  1027-1043 | ([Manz et al., 1992](#_ENREF_4)) |
